# Aging Hallmarks: The Benefits of Physical Exercise

**DOI:** 10.3389/fendo.2018.00258

**Published:** 2018-05-25

**Authors:** Alexandre Rebelo-Marques, Adriana De Sousa Lages, Renato Andrade, Carlos Fontes Ribeiro, Anabela Mota-Pinto, Francisco Carrilho, João Espregueira-Mendes

**Affiliations:** ^1^Faculty of Medicine, University of Coimbra, Coimbra, Portugal; ^2^Clínica do Dragão, Espregueira-Mendes Sports Centre – FIFA Medical Centre of Excellence, Porto, Portugal; ^3^Dom Henrique Research Centre, Porto, Portugal; ^4^Endocrinology, Diabetes and Metabolism Department, Coimbra Hospital and University Center, Coimbra, Portugal; ^5^Faculty of Sports, University of Porto, Porto, Portugal; ^6^3B’s Research Group—Biomaterials, Biodegradables and Biomimetics, University of Minho, Headquarters of the European Institute of Excellence on Tissue Engineering and Regenerative Medicine, Guimarães, Portugal; ^7^ICVS/3B’s–PT Government Associate Laboratory, Guimarães, Braga, Portugal; ^8^Orthopaedics Department of Minho University, Minho, Portugal

**Keywords:** aging, physical exercise, hallmarks, cellular, molecular, life span

## Abstract

World population has been continuously increasing and progressively aging. Aging is characterized by a complex and intraindividual process associated with nine major cellular and molecular hallmarks, namely, genomic instability, telomere attrition, epigenetic alterations, a loss of proteostasis, deregulated nutrient sensing, mitochondrial dysfunction, cellular senescence, stem cell exhaustion, and altered intercellular communication. This review exposes the positive antiaging impact of physical exercise at the cellular level, highlighting its specific role in attenuating the aging effects of each hallmark. Exercise should be seen as a polypill, which improves the health-related quality of life and functional capabilities while mitigating physiological changes and comorbidities associated with aging. To achieve a framework of effective physical exercise interventions on aging, further research on its benefits and the most effective strategies is encouraged.

## Introduction

Aging is a complex and intraindividual process, usually defined as a time-dependent progressive loss of the individual’s physiological integrity, which eventually leads to deteriorated physical function (1). Within this line, the accumulated molecular and cellular damage across the individual’s life span often leads to age-associated pathological conditions and thus makes them more prone to death (2–5). Understanding the specific cellular and molecular mechanisms implicit in aging still represents one of the most complex and integral issues that biological research has yet to overcome.

**Figure 1:**
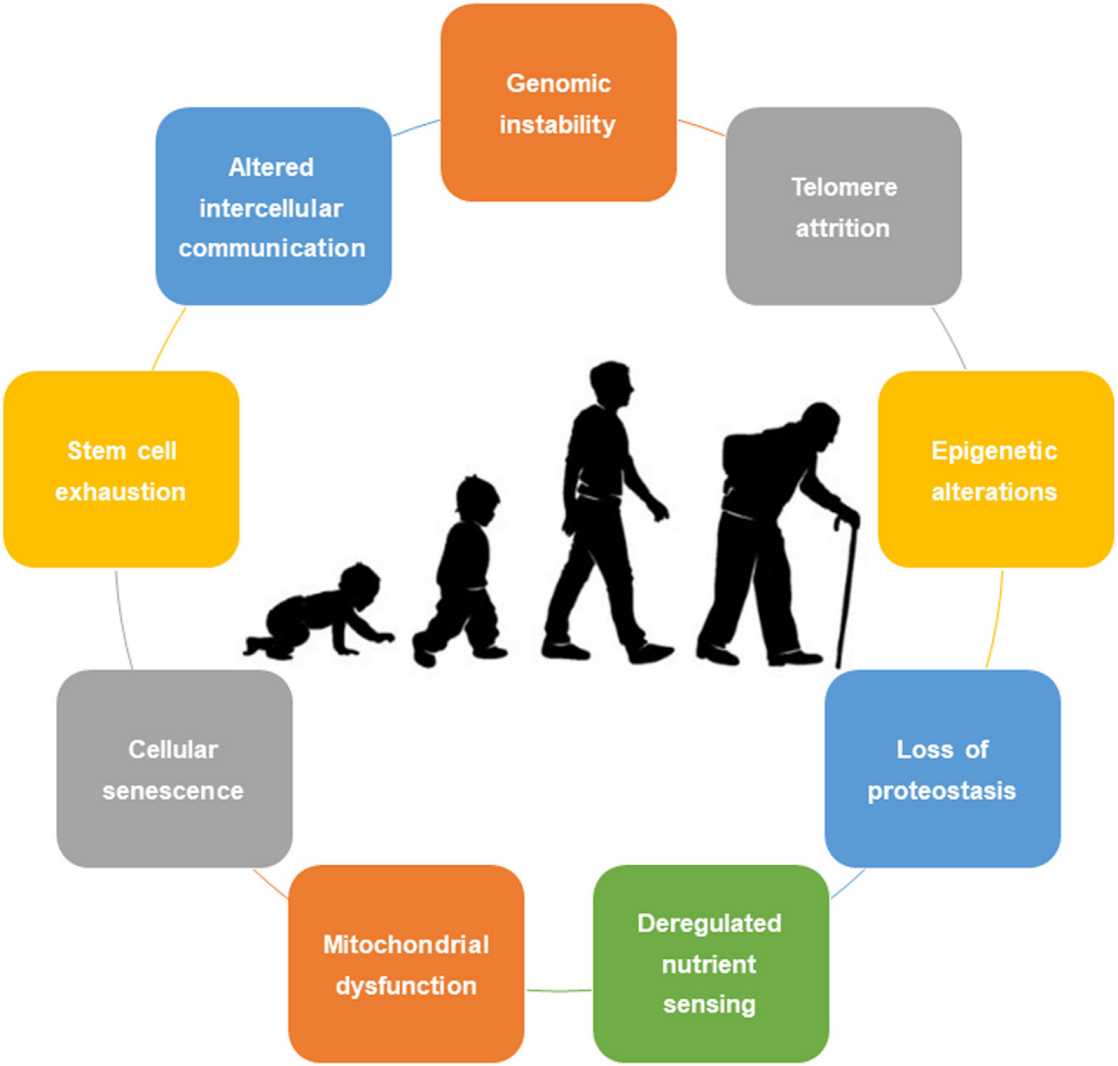
Nine cellular hallmarks contributing to aging.

Despite hundreds of explored and developed theories, not a single one fully and comprehensively explains the process of aging (6). Traditionally, aging was not seen as an adaptation or genetically programmed phenomenon. More recently, biologic currents point to two main theories: the programmed aging and the damage or error-based theories. The first suggests an intrinsic biologic programmed deterioration of the structural and functional capacity of the human cells (7). The latter highlights the cumulative damage to living organisms leading to intrinsic aging (8). Nonetheless, a combination of these theories is usually preferred. In this sense, López-Otín et al. (1), in a state-of-the-art review, proposed nine cellular and molecular hallmarks that contribute to the process of aging, including (1) genomic instability, (2) telomere attrition, (3) epigenetic alterations, (4) loss of proteostasis, (5) deregulated nutrient sensing, (6) mitochondrial dysfunction, (7) cellular senescence, (8) stem cell exhaustion, and (9) altered intercellular communication (Figure 1). These hallmarks should be expressed during normal aging, with their experimental aggravation speeding up the aging process, and in contrast, their experimental amelioration retards the normal aging process, thus increasing a healthy life span.

The world population has been progressively aging and thus raising the average life expectancy. In 2015, an estimated 8.5% (617.1 million) of the world population were aged 65 and older. Within this line, the older population percentage is expected to keep increasing, with an average annual increase of 27.1 million, representing 12 and 16.7% of world population by the years 2030 and 2050, respectively (9). With this life span increase and its associated aging comorbidities, a growing challenge has arisen to make older people physically active and functionally independent until the rest of their lives. Along with the nine cellular and molecular hallmarks stated above, aging is known to be correlated with several cardiovascular, cardiorespiratory, musculoskeletal, metabolic, and cognitive impairments (10). In this sense, regular physical activity in the older population—especially aerobic and resistance training—plays an important role at a multisystem level, preventing severe muscle atrophy, maintaining cardiorespiratory fitness and cognitive function, boosting metabolic activity, and improving or maintaining functional independence (10–13). Within this line, Garatachea et al. (10) and Nelson et al. (13) made an outstanding summary of the specific antiaging effects of physical exercise at the multilevel system, including (1) increasing neurogenesis and attenuating neurodegeneration and cognitive alterations; (2) decreasing blood pressure levels and increasing numerous cardiovascular functions, such as maximal cardiac output, regional blood flow and blood volume, body fluid regulation, endothelial and autonomic function, vagal tone and heart rate variability, and cardiac preconditioning; (3) improving respiratory function by increasing ventilation and gas exchange; (4) enhancing metabolic function in raising the resting metabolic rate, muscle protein synthesis, and fat oxidation; and (5) augmenting muscle function and, subsequently, body composition by improving muscle strength and endurance, maintaining or regaining balance, motor control, and joint mobility, as well as reducing weight and regional adiposity and increasing muscle mass and bone density. In addition, physical exercise has a positive antiaging impact at the cellular level, and its specific role in each aging hallmark is described below.

## Effect of Physical Exercise on Each Cellular Hallmark

### Genomic Instability

The accumulation of genetic damage throughout one’s life span takes a major role in the aging process (14). Genomic instability, caused by exogenous (physical, chemical, and biological) and endogenous factors [deoxyribonucleic acid (DNA) replication errors, spontaneous hydrolytic reactions, and reactive oxygen species (ROS)], often results in mutations, translocations, chromosomal gains and losses, telomere shortening, and gene disruption (15). In this sense, several cellular events contribute to genomic instability and subsequently to aging, including somatic mutations of nuclear DNA, mutations and deletions in aged mitochondrial DNA (mtDNA), and defects in the nuclear lamina (14, 16, 17). Increased genomic damage has been linked to aging, highlighted by the DNA repair deficits found in accelerated mice models, translated into many human progeroid syndromes (15, 18, 19). Likewise, mtDNA mutations in aged subjects appear to be induced by early replication errors rather than later cumulative oxidative damage (20). In addition, the mutation of the nuclear lamina protein gene encoding and disturbances on its maturation or dynamics leads to a few progeria syndromes (21–23). This premature aging is supported by the delayed onset of progeroid features and extended life span after decreasing prelamin A or progerin levels (24–26).

In the face of genomic instability, the organism has developed a panoply of DNA repair mechanisms that skirmish altogether to overcome DNA nuclear damage (27). Within the same line, genomic stability systems hold specific mechanisms to maintain proper telomere length and function and mtDNA integrity (28, 29). Pharmacological and biologic strategies (mainly hormonal or genetic therapies) have been developed (30–32); howbeit, further research is required to validate nuclear architecture reinforcement for delaying normal aging (1).

Exercise plays a role in maintaining genomic stability. In rodent models, aerobic exercise improves DNA repair mechanisms and nuclear factor kappa B (NF-kB) and peroxisome proliferator-activated receptor gamma coactivator 1-alpha (PGC-1α) signaling (33–35). Moreover, it augments DNA repair (36) and decreases the number of DNA adducts (up to 77%), related to aging and several risk factors for cardiovascular diseases (37). In addition, a six-month resistance training program in an institutionalized elderly population showed a tendency to reduce cell frequency with micronuclei (~15%) and the total number of micronuclei (~20%), leading to a higher resistance against genomic instability (38). In a meta-analysis comprising data of 478 genetic elements (387 unique genes) associated with exercise from 1,580 individuals, 238 out of 387 genes decreased in DNA methylation percentage after physical exercise among older people. More specifically, the genes that presented DNA methylation decreases were associated with a cancer-suppressing micro-ribonucleic acid (miRNA) gene network (39).

### Telomere Attrition

Telomeres are ribonucleoprotein complex structures that protect the integrity of information-carrying DNA throughout cell cycle, preventing the base pair loss of chromosomal DNA during cellular division. Over consecutive cellular divisions, telomere length naturally decreases till a minimum critical size, which precludes further cell division, causing cellular senescence or apoptosis, also known as the end replication problem (40). Telomerase, as an enzyme with a catalytic unit, answers to the end replication problem, promoting telomere lengthening (41). Nevertheless, the fact that most mammalian somatic cells did not express this enzyme explains the progressive loss of the terminal chromosomes’ ends as well as the limited proliferative capacity observed in some *in vitro*-cultured cells (42, 43). This enzyme deficiency in humans has been linked to premature manifestations of chronic diseases specially related to scarce tissue regenerative ability (such as pulmonary fibrosis, congenital dyskeratosis, or aplastic anemia) (44). Telomere shortening is described during normal aging in human and mice cells (45–50). The fact that telomere length decreases with aging, contributing to the normal cell senescence process, suggested that this could be a potential marker for biological aging (51). Moreover, the leukocyte telomere length is also positively associated with a number of healthy living years, associated with numerous chronic conditions and their complications and with the mortality risk mainly at younger ages (52, 53). The association of chronic inflammation and elevation of pro-inflammatory cytokines [cytokine tumor necrosis factor (TNF-α) and interleukin (IL)-6] and shortened leukocyte telomeres has been proposed by several authors (54–57). Regarding TNF-α, this cytokine seems to have a specific role in downregulating telomerase activity, causing telomere shortening (55).

Interestingly, recent evidence supports that telomerase activation can revert aging, namely, in the premature aging of telomerase-deficient mice when the enzyme is genetically reactivated (58). The relationship between physical activity and healthy aging is well recognized, but the association between physical activity and telomere length remains unclear. Aging induces DNA damage accumulation, especially in some particularly sensitive chromosomal regions such as telomeres, and recent data suggest that physical activity may play a protective role against stress-related telomere attrition (28, 59). Although the potential mechanism is unclear, exercise exhibits a favorable impact on telomere length, especially on a chronic pattern and particularly in older individuals antagonizing the typical age-induced decrements in telomere attrition. Several potential mechanisms have been discussed linking exercise and telomere length decrements to changes in telomerase activity, inflammation, oxidative stress, and decreased skeletal muscle satellite cell content (60).

Exercise has been associated with an upregulation of protective proteins (such as telomeric repeat-binding factor 2) and DNA repair pathway proteins (such as Ku proteins) as well as a downregulation of negative regulator proteins of cell cycle progression (such as p16) in middle-aged athletes supporting this relationship (61). Additionally, although it increases oxidative stress, continuous physical exercise is associated with antioxidant activity and inferior levels of ROS, favoring the REDOX balance, protecting from DNA damage and subsequently shorter telomere attrition (62–64).

Satellite cells are specific skeletal muscle cell precursors activated during muscle regeneration processes or in response to muscle injuries. A positive correlation exists between the number of satellite cells and skeletal muscle telomere length in older adults (61). This pool of cells decreases normally during aging and appears to connect physical activity and skeletal muscle preservation since both resistance and aerobic exercise act as satellite cell pool stimulators, equalizing the decline related to aging (65).

### Epigenetic Alterations

The relationship between epigenetic regulation and aging is controversial and complex (1). Epigenetics studies the mitotically and/or meiotically heritable changes within genetic function that cannot be explained by DNA sequence changes (66). Although *epigenome* refers to the combination of chemical changes to DNA and histone proteins in a cell, epigenetic changes include alterations in DNA methylation patterns, the posttranslational modification of histones, and chromatin remodeling (such as miRNA expression changes) (67).

A multiplicity of epigenetic modifications affects all tissues and cells throughout life (68). Increased histone H4K16 acetylation, H3K4 trimethylation, or H4K20 trimethylation, as well as decreased H3K27 trimethylation or H3K9 methylation, make up age-associated epigenetic marks (69, 70). In mammals, at least three members of the sirtuin (SIRT) family—SIRT1, SIRT3, and SIRT6—contribute to healthy aging (71–73). For example, the transgenic overexpression of mammalian SIRT1 (closest homolog to invertebrate Sir2) improves health aspects during aging despite not increasing longevity (73). Losing the function of SIRT6 reduces longevity, and the gain extends longevity in mice (71, 74).

Actually, the literature clearly reveals that the epigenetic response is highly dynamic and influenced by different environmental and biological factors, such as aging, nutrient availability, and physical exercise. Regular aerobic exercise can change the human genome through DNA methylation (75). Transient hypoxia conditions are a good example (76). Thus, by using epigenetic mechanisms, aerobic exercise can induce the transcription of genes encoding telomere-stabilizing proteins and telomerase activity not only in animals (61, 77, 78) but also in humans (61). Losing promoter methylation and histone H4 deacetylation is associated with the changed gene expression profile in adaptation to aerobic exercise (63–67, 75–79). Also, the class II HDACs 4 and 5 (transcriptional repressors) can translocate from the nucleus to the sarcoplasm of muscle fibers in response to aerobic exercise (79). In human and mouse muscles, the mitochondrial transcription factor (TFAM), PGC-1a methylation, citrate synthase, MEF2A, gene promoters, and pyruvate dehydrogenase kinase isozyme (PDK4) decrease after acute aerobic exercise (80). In addition, aerobic exercise-induced SIRT-1 regulates the tumor suppressor PGC-1a, p53, NF-jB, and other transcription factors *via* its deacetylase activity (3, 81). Exercise effects are blocked by the overexpression of HDAC5 in transgenic mice, suggesting that histones are important in the transcriptomic response to muscle contraction (82).

Acute exercise is also associated with increases in messenger RNA (mRNA) expression by transient DNA hypomethylation of gene-specific promoter regions (76, 81). In turn, during the recovery period, this mRNA elevation enables protein synthesis and induces gradual structural remodeling and long-term functional modifications (83). Also, calcium and insulin signaling was recently found to be differentially methylated in skeletal muscle after aerobic exercise (83). As far as we know, physical exercise, either aerobic or resistance, can influence miRNAs’ histone modifications or DNA methylation in various tissues. These adaptations can occur in at least the brain, muscle, or cardiovascular system and are intrinsic to the skeletal muscle response during exercise (e.g., mitochondrial respiratory capacity, substrate delivery, and contractile function) (84, 85).

Chronic moderate aerobic exercise increases the methylation levels of the pro-inflammatory apoptosis-associated speck-like protein caspase gene, which modulates IL-18 and IL-1b in the aged leukocytes, thereby contributing to reduced age-related pro-inflammatory cytokines (86). In addition, several myogenic regulatory factors—e.g., myoblast determination protein 1, myogenin, or myogenic factors 5 (Myf5) and 6 (Myf6, also known as myogenic regulatory factor 4, Mrf4, or herculin)—can help fight age-related sarcopenia and frailty, all of them modulated by aerobic or resistance exercise (87–89). Exercise can also upregulate brain-derived neurotrophic factor (BDNF) induction and promote the remodeling of the chromatin containing the BDNF gene (90).

The effect of physical exercise on epigenetic changes is just the beginning. However, studies so far show that an important modulation of exercise exists on the epigenetics mechanisms, particularly in DNA methylation, specially of regular physical exercise.

### Loss of Proteostasis

Aging and some aging-related diseases are linked to impaired protein homeostasis, also known as proteostasis (91). The array of quality control is guaranteed through distinct mechanisms that involve location, concentration, conformation, and the turnover of individual proteins, such as autophagy, proteasomal degradation, or chaperone-mediated folding (92). These functions prevent the aggregation of damage components and ensure the continuous renewal of intracellular proteins, degrading altered proteins.

A loss of function or incoordination of these processes leads to accumulated damaged proteins and thus aging-associated deleterious effects (93) and neurological age-related conditions such as Alzheimer’s or Parkinson’s disease (91).

Aging impairs the autophagy–lysosomal and ubiquitin–proteasome systems, which play a central role in cellular proteostatic mechanisms (94, 95). Conversely, physical activity induces brain, muscle, and cardiac autophagy (96). A joint program of moderate-intensity leg-resistance exercises and walking demonstrated to upregulate autophagy muscle markers in old women (97), despite that these data are still restricted to aged subjects, and scarce evidence is still available in humans.

In mouse models, this effect seems to be mediated by the activation of BCL-2–beclin 1 complex (98). Acute resistance exercise programs induced muscle protein synthesis and decreased protein degradation through the activation of class 3 phosphatidylinositol 3OH kinase Vps34 mVps34, which forms an autophagy regulator complex with beclin-1 (99, 100). Moreover, in transgenic mice, the beclin-1 disruption reduces autophagy, leading to neurodegeneration (101).

Aerobic exercise induces autophagy, thus preventing the loss of strength and muscle mass through the modulation of IGF-1, protein kinase B (Akt)/mammalian target of rapamycin (mTOR), and Akt/Forkhead box O3A signaling pathways (102, 103), decreasing cardiovascular disease risk (97) and eliminating damaging proteins triggering neurodegeneration (98).

Similarly, the aging human brain exhibits a downregulation of beclin-1 (104). Higher basal levels of autophagy were related to healthy human exceptional longevity, and healthy centenarians have higher serum levels of beclin-1 compared with young controls (105).

Atrogin-1 (MAFbx) is a muscle-specific ubiquitin ligase involved in muscle atrophy through FoxO signaling (106). The atrogin-1 upregulation is associated with cardiac and skeletal muscle atrophy, and atrogin-1 knockout mouse models corroborate its association with autophagy dysfunction, cardiomyopathy, and premature death (107, 108). Within the same line, comparing aged atrogin-1 knockout mice with age-matched controls, the former shows a reduced tolerance to treadmill exercise and shortened life span (106).

The synthesis of cytosolic and organelle-specific chaperones is impaired in aging (109). Thus, chaperone associated functions, such as folding and protein stability, are conditioned throughout one’s life span (110, 111). In animal models, the upregulation of cochaperone of the heat-shock proteins (HSPs) was associated with prolonged life-span phenotypes (112), and HSF-1 activation, the heat-shock response regulator, was linked to longevity and thermotolerance (113, 114). Despite limited comparison studies, evidence supports that acute endurance- and resistance-type exercise protocols are associated with increased HSPs transcription not only during activity but also immediately postexercise or several hours following exercise, which points out the possible favorable impact of physical activity on proteostasis (115).

### Deregulated Nutrient Sensing

The growth hormone (GH) is produced by the anterior pituitary gland and is regulated by the growth hormone-releasing hormone, acting mainly in the hepatocytes to induce insulin-like growth factor 1 (IGF-1) secretion. IGF-1 is also produced in distinct tissues, such as osteocytes, chondrocytes, and muscle, to act in an autocrine or paracrine pattern (116).

Insulin and IGF-1 share the same intracellular signaling pathway, an important aging-controlling route highly conserved during evolution. In this sense, enhanced longevity has been associated with the reduced functions of GH, IGF-1, and insulin receptors and their intracellular effectors (such as Akt and mTOR complexes) (117–119). Within this scope, several authors associated dietary restriction with an increased life or health span probably mediated by an attenuation of insulin and IGF-1 signaling pathway (118, 120–132).

Regarding the intracellular effectors downstream, in animal models, the transcription factor FOXO represents the most relevant alteration linked to longevity (123, 124). The tumor-suppressor gene PTEN has also been associated with an antiaging impact on this signaling pathway, promoting energy expenditure and improving mitochondrial oxidative metabolism (125, 126). This balance between molecules with antiaging properties, emphasized by nutrient scarcity [FOXO, 5’-adenosine monophosphate-activated protein kinase (AMPK), and PTEN] against those that favor the aging process (GH, IGF-1, Akt, and mTOR), shows the relevance of deregulated nutrient sensing as a hallmark of aging (123, 124, 127).

Aging is also physiologically associated with somatopause, which represents a progressive decline in the GH secretory rate starting in the third decade of life, as reflected in decreasing IGF-1 levels (117, 128). In mouse models of premature aging, this GH-IGF-1 axis decline is also noted, highlighting this common denominator in normal and sped-up aging processes (129). This paradoxical observation can be integrated as a defensive response that downmodulates the GH-IGF-1 axis, promoting lower cell growth and metabolism, reducing cellular damage and aiming to extend life span (130).

Besides the glucose sensing related to GH-IGF-1 axis activity, the interest on other nutrient-sensing systems is increasing—mTOR, amino acid concentrations and anabolic metabolism; AMPK and adenosine monophosphate levels; and SIRTs and NAD+ levels. These last two nutrient sensors, AMPK and SIRTs (SIRT 1–7), arise as alternative markers to low-energy states opposite mTOR (117, 131, 132).

Exercise plays an important role in not only the glucose-sensing somatotrophic axis but also the three nutrient-sensing systems referred above, promoting a beneficial anabolic cellular state (133–136). The effect of exercise on glucose metabolism through increased glucose transporter type 4 production is another well-known mechanism of improved insulin sensibility associated with physical activity (137). Additionally, exercise-induced GH and IGF-1 levels are influenced by exercise intensity, duration, and type (higher in intense interval protocols and resistance exercise) (138–140). Thus, the increased muscle protein synthesis associated with resistance exercise is pointed out as a successful strategy to prevent age-related sarcopenia (141–143).

### Mitochondrial Dysfunction

The clear causal relationship between mitochondrial dysfunction and aging has long been a target of great discussion; however, the specific mechanisms involved remain unrevealed. Initially, the mitochondrial free radical aging theory proposed that with increasing age came a progressive mitochondrial dysfunction, increasing ROS levels and subsequently further mitochondrial deterioration and generalized cellular damage (144). Nevertheless, dysfunctional mitochondria may contribute to the increase in aging process, independent of ROS levels (145, 146). Interestingly, increased ROS levels may extend the life span of yeast and *Caenorhabditis elegans* (147–149). In addition, a genetically manipulated impaired mitochondrial function that does not increase ROS levels does not seem to accelerate the aging process (145, 146, 150–152). Within the same lines, mice with genetically manipulated increased ROS levels, greater oxidative damage, or higher antioxidant defense levels do not seem to age quickly or possess extended life spans (145, 146, 150–154). In fact, the main ROS effect is to activate compensatory homeostatic responses. However, with the increased cellular stress and damage present in the aging process, the ROS levels, when exceeding a determined threshold, may even deepen age-related damage (155). Altogether, the findings led to the reconsideration of the ROS role in aging (156).

With increasing age comes a decline in mitochondrial integrity and biogenesis because of alterations in mitochondrial dynamics and mitophagy inhibition, impairing dysfunctional mitochondria removal (156). Several mechanisms seem to be related to mitochondrial integrity and biogenesis, including mitochondrial deficiencies that increase their predisposition to permeabilize in the presence of stress, resulting in activated ROS-mediated and permeabilization-facilitated inflammatory reactions (157, 158). Within this line, lower biogenesis and reduced clearance often lead to a combination of increased damage and reduce mitochondrial turnover, which also accelerate the aging process (1).

With the aging process comes an accumulation of many mtDNA mutations, mostly deletions, which affect many tissues, including nervous and skeletal muscle tissues (159–162). Within this line, the respiratory system efficacy declines with increasing age, which leads to increased electron leakage and lower adenosine triphosphate generation levels (163). The accumulation of oxidative stress-induced mtDNA mutations leads to the progressive decay of the mitochondrial function (164, 165). Moreover, mtDNA is more vulnerable to oxidative damage than nuclear DNA as it lacks histones protection, DNA repair capacity, and non-coding introns (166). Additionally, mtDNA damage may proliferate as cells multiply, resulting in expanded physiologic damage (167).

The regular practice of physical exercise has a positive impact in the mitochondrial function. In this sense, endurance-trained humans presented higher levels of mitochondrial proteins expression, mtDNA, and TFAMs (168). When considering sedentary mtDNA mutator mice, which displayed symptoms of accelerated aging, a 5-month aerobic exercise program induced systemic mitochondrial biogenesis in the mtDNA and increased multiorgan oxidative capacity, thus providing phenotypic protection and reducing multisystem pathology and the risk for premature mortality (169). Hence, regular physical exercise may maintain a pool of bioenergetically functional mitochondria that, by improving the systemic mitochondrial function, contribute to morbidity and mortality risk reduction throughout one’s life span (169–172). Similarly, in the elderly population, the resistance exercise through the PGC-1 and SIRT regulators (173) has decreased DNA oxidative damage (by stimulating their endogenous antioxidant defenses) (174), mitochondrial alterations induced by aging (37), and the improved oxidative capacity of muscle fibers (175).

Aged individuals often show a deficiency in cytochrome c oxidase within the sarcopenic skeletal muscles’ fibers with higher levels of mtDNA mutations (176–180). Moreover, the decreased mitochondrial enzyme activity frequently seen in aged individuals (181, 182) is accompanied with a downregulation of mRNAs encoding mitochondrial proteins (183–185). In this sense, resistance training has the potential to shift the mtDNA of skeletal muscle from healthy aged individuals, augmenting the mitochondrial function (180). Within the same line, a 6-month resistance exercise-training program reversed aging transcriptional signature levels approaching the ones from younger adults, thus enhancing the mitochondrial function (185).

### Cellular Senescence

Cellular senescence is the stable arrest of the cell cycle combined with stereotyped phenotypic modifications (186–188). Initially, cellular senescence was associated with telomere attrition, but other age-related triggers were posteriorly identified, namely, non-telomeric DNA damage, and derepression of the INK4/ARF locus (42, 187, 189). Some authors have directly used quantification of senescence-associated β-galactosidase (SABG) to identify senescent cells in different-aged tissues (190, 191). Surprisingly, they identified higher SABG values in hepatic, skin, lung, and spleen tissues in older mice compared to younger mice. However, this differential was not observed in heart, skeletal muscle, and kidney tissues. Based on these facts, the concept that cellular senescence is not a generalized property of all tissues in aged organisms was raised (191). Beyond that, senescent cell accumulation in different tissues seems to be dependent, in one hand, on an increased rate of senescent cell generation and, in other hand, on a decreased rate of clearance (192–194).

The cell’s senescence process is usually associated with a deleterious purpose from senescent cell proliferation with aging. Nevertheless, its primary purpose is to prevent damaged cell proliferation and trigger their demise by the immune system, resulting in a beneficial cell compensatory response, contributing to tissue homeostasis. When tissues exhaust their regenerative capacity, the compensatory response to damage becomes harmful and accelerates aging (195).

Exercise, specifically aerobic, induces the secretion of antitumorigenic myokines and greater natural killer cell activity, contributing to a decreased incidence of oncologic disease and improved cancer prognosis (196). Moreover, the increased expression of telomere repeat-binding factor 2 and Ku70 and the reduced expression of apoptosis regulators (such as cell cycle-checkpoint kinase 2, p16INK4a, and p53 or survival regulators) are associated with the beneficial impact of exercise on cellular senescence (61).

A part of DNA damage, excessive mitogenic signaling, is strongly associated with the senescence (197). The main mechanisms reported that implement senescence in response to this variety of oncogenic insults were p16INK4a/Rb and p19ARF/p53 pathways (1, 198). Senescent cells present upregulated p16INK4a and p21 cell cycle inhibitors. p21 is a downstream target of p53 and telomere dysfunction (199, 200). Aerobic exercise has been inversely correlated with p16INK4a mRNA levels in peripheral blood T lymphocytes, which might promote protective outcomes against age-dependent alterations (201).

The secretome of a senescent cell presents dramatic alterations becoming particularly enriched in pro-inflammatory cytokines and matrix metalloproteinases that may contribute to aging (10, 202, 203). This senescence-associated secretory phenotype (SASP) as a DNA damage response explains how senescent cells alter tissue microenvironments (204). Once again, aerobic exercise suppresses liver senescence markers and downregulates inflammatory mediators (reducing gamma glutamyltranspeptidase activity and levels of p21, p53, and IL-6) (205).

Exercise is capable of upregulating cardiac telomere-stabilizing proteins, providing protection against doxorubicin-induced cardiomyopathy and promoting antisenescent effects (77). Within the same line, the same research group (61) showed that besides improving telomere biology in the thoracic aorta and in mononuclear cells, exercise could also reduce the vascular expression of apoptosis regulators. Moreover, it allows endurance athletes to increase telomerase activity and downregulate cell-cycle inhibitors compared with sedentary individuals. In addition, Song et al. (206) found that in humans, the practice of aerobic exercise reduced the expression of DNA damage biomarkers and correlated negatively with telomere length in peripheral blood T lymphocytes and positively with p16INK4a expression, supporting previous findings.

### Stem Cell Exhaustion

In aging, the declining regenerative potential of tissues is obvious (1). A good example is the age-related decline in hematopoiesis, causing a diminished production of adaptive immune cell, a process designated as immunosenescence (207). Similar processes were found in adult stem cell compartments, including the mouse forebrain (208), muscle fibers (209), or bone (210). An overall decrease in cell-cycle activity of hematopoietic stem cells has been revealed (211), connected with the overexpression of cell cycle-inhibitory proteins such as p16INK4a (212) and DNA damage accumulation (211).

For the long-term maintenance of the organism, the deficient proliferation of stem and progenitor cells is harmful, but an excessive proliferation can also be deleterious by speeding up the exhaustion of stem cell niches (1). Within this line, physical exercise is one of the most potent stimuli for the migration/proliferation of the stem cell subsets from their home tissue to impaired tissues for later engraftment and regeneration (95). In this sense, regular physical exercise attenuates age-associated reduction in the endothelium reparative capacity of endothelial progenitor cells (213). In addition, exercise activates pluripotent cells’ progenitors, including mesenchymal and neural stem cells, which improve brain regenerative capacity and cognitive ability (95).

The stem cells more affected by aging are myogenic, known as satellite cells (214). Satellite cell alterations manage the reduced replacement and repair efficiency potential in human skeletal muscle tissue myofibers. Age-reduced functionality or the number of these cells inhibits proper muscle-mass maintenance (214–217). Age-related atrophy by type II muscle fibers is influenced by the decline in the content of type II muscle fiber satellite cells (214). Sarcopenia is related to these cells’ atrophy and so are its pathophysiological mechanisms (218). Besides that, aging reductions in strength and muscle mass are directly connected with myonuclear content, the muscle fiber type-specific cross-sectional area, and satellite cell content (219). Animal studies showed that aerobic exercise not only promotes satellite cell pool expansion in young and old mice (220) but also potentiates myofibers with greater numbers of satellite cells in young and old rats (221). Thus, skeletal muscle regulation depends on the satellite cells (222). This contribution to skeletal muscle regeneration is well documented (223, 224) and involves several mechanisms, including neurotrophic and vascular factors (IGF-1 and other growth factors), immune response, neurotransmitters, and cytokines (such as IL-6, testosterone, or nitric oxide), most of which are modulated by exercise (167). Resistance training can induce, in young adults and during aging, the hypertrophy of type II fibers (225, 226) by skeletal muscle satellite cell proliferation and differentiation, which attenuates prosarcopenic physiological and age-related events (214, 218, 219). Moreover, muscular hypertrophy generated by resistance exercise training is related to satellite cells (227). Myostatin, a protein that inhibits muscle differentiation and growth in the myogenesis process, is also involved in the process (228). Probably, the same localization of myostatin and satellite cells explains the worsened myogenic capacity of the aged skeletal muscle (229). In fact, Snijders et al. (228) revealed an aging-blunted activation of type II muscle fiber satellite cells in response to acute stimuli of resistance exercise.

Interestingly, pharmacological interventions are also being explored to increase stem cell function. Using rapamycin for the inhibition of mTORC1 can delay aging by improving proteostasis and affecting energy sensing, which may improve stem cell function, in the hematopoietic system and intestine (230–232). The pharmacological inhibition of the GTPase CDC42, whose activity is increased in aged hematopoietic stem cells, may also rejuvenate human senescent cells (233).

### Altered Intercellular Communication

The physiological aging process implicates several alterations on intracellular communication mechanisms, namely, in neuroendocrine, endocrine, and neuronal levels (234–237). Inflammation plays a central role in this age-related alteration, contributing to a predominant pro-inflammatory phenotype associated with progressive aging, also known as “inflammaging” (238). This inflammaging is caused by distinct mechanisms: pro-inflammatory tissue damage accumulation, additional cumulative dysfunction of the immune system, elevated levels of pro-inflammatory cytokines’ secretion by senescent cells, altered autophagy response, and the increased activation of the NF-kB transcription factor (163, 238–240). All these mechanisms will promote the activation of different pro-inflammatory pathways leading to increased levels of IL-1b, TNF, and interferons (163, 238). In this sense, different endocrine axes (renin–angiotensin, adrenergic, and insulin-GH), as part of neurohormonal signaling, are altered with aging because of increased inflammatory reaction levels, the decline of immunosurveillance against pathogenic agents and premalignant cells, and composition changes in the peri- and extracellular environment (238). Additionally, the decay factor AU-binding factor 1 (AUF1 or heterogeneous nuclear ribonucleoprotein D) has also been linked to inflammaging as an important factor in inflammatory response cessation, conditioning cytokine mRNA degradation, and contributes also to maintaining telomere length by activating the expression of the telomerase catalytic subunit telomerase reverse transcriptase, linking this same factor to different hallmarks discussed above (241). Chronic muscle contractile activity upgraded different AUF1 isoforms secretion (p37, p40, and p45) in the muscle of healthy rats, resulting in improved muscle plasticity (242).

Pro-inflammatory state and stress activate hypothalamic NF-kB expression, which downregulates gonadotropin-releasing hormone neurons and subsequently gonadotropins by the anterior pituitary, explaining some age-related comorbidities such as bone fragility, muscle weakness, and reduced neurogenesis (237). The enhanced activation of the NF-kB transcription factor is referred to as one of the transcriptional signatures of aging, and its expression restriction has been associated with skin rejuvenation in animal models (243). Besides, the use of genetic or pharmacological inhibitors of the NF-kB signaling was associated with the prevention of age-associated features in distinct sped-up aging mouse models (32, 244). The SIRT family (SIRT 1–7), in the same point of view, seems to downregulate inflammation-related genes acting as a protective factor to aging and many other inflammatory pathological conditions (245–248).

The cross-talk interorgan may explain the correlation between aging-related changes in different tissues. Cellular senescence influences neighbor cells during aging *via* gap–junction contacts, growth factors, interleukins, and ROS, highlighting the importance of microenvironment contribution during the process and offering the possibility to modulate aging in different levels (249). Chronic exercise, especially aerobic type, may restore defective intercellular communication, decreasing mitochondrial ROS production and upregulating the endogenous antioxidant profile (33).

Muscle contraction is traditionally associated with myokine secretion (proteins, growth factors, cytokines, or metallopeptidases) elevated during and after exercise (97). Interestingly, the muscle-released IL-6 creates a healthy influence, inducing the production of anti-inflammatory cytokines, IL-1 receptor antagonist, IL-10, or TNF soluble receptors, while restraining pro-inflammatory cytokine TNF-α production (250, 251). Within these lines, several authors associated lifelong aerobic exercise training with lower inflammatory levels, especially with lower levels of C-reactive protein, IL-6, and TNF-α, particularly in advanced decades of life (252–254).

## Physical Activity Recommendations

Older individuals must practice physical exercise to maintain the health-related quality of life and functional capabilities that mitigate physiological changes and comorbidities associated with aging. Recommendations made herein are based on the most recent American College of Sports Medicine Guidelines (255) (Table 1). Physical exercise should include aerobic exercise, muscle strengthening and endurance training, and flexibility and neuromotor exercises. Physical exercise difficulty and intensity progression should be tailored to the individual’s tolerance, preference, and specific needs. Thus, lighter intensity and duration are recommended at earlier stages, especially for those who are deconditioned or functionally impaired or present chronic conditions that preclude the performance of more demanding physical tasks. More debilitated and frail individuals may initially require aerobic training activities to improve their physical fitness before proceeding to more demanding tasks. In opposition, individuals presenting sarcopenic muscles may need to improve their muscular strength and endurance before engaging in aerobic training. When the chronic conditions or comorbidities preclude accomplishing the recommended minimum of physical exercise, older individuals should not remain sedentary, and physical training should be performed as tolerated to provide a therapeutic benefit. Training sessions should be supervised by a qualified health professional and finish with an adequate cooldown (gradual reduction of physical intensity complemented with flexibility exercises), especially among individuals with cardiovascular disease.

**Table 1:** General guidelines for exercise prescription in older individuals.

Type of exercise	Frequency	Intensity (0–10)	Duration/volume
Aerobic exercise	≥5 days/week for moderate intensity	5–6	30–60 min/day in bouts of at least 10 min each to total 150–300 min/week
	≥3 days/week for vigorous intensity	7–8	20–30 min/day to total 75–100 min/week
	3–5 days/week for combination of moderate and vigorous intensity	5–8	Combination of both
Muscle strengthening	≥2 days/week	Start with light (40–50% of 1RM)	8–10 exercises involving the major muscle groups with 1 set of 10–15 repetitions each
		Progress to moderate (60–70% of 1RM)	
Flexibility	≥2 days/week	Until tightness or slight discomfort	Hold for 30–60 s

*For quantifying the perceived physical exertion in older individuals, a 10-point scale should be used: 0 is considered an effort equivalent to sitting and 10 an all-out effort; moderate intensity comprises 5 and 6 and vigorous intensity between 7 and 8. Moderate intensities should produce a noticeable increase in heart rate and breathing, while a vigorous intensity should produce a substantial increase in heart beat and/or breathing (82)*.

Individuals with increased fall risk or present functional and mobility limitations benefit from the addition of neuromotor exercise training (2–3 days/week), comprising balance, agility, and proprioceptive training (255–257). General recommendations to progressively increase the exercises’ difficulty include (i) more challenging postures by gradually shortening the support base, (ii) more exercises comprising more dynamic movement that perturb the center of gravity, (iii) higher focus on the postural muscle groups exercises, and (iv) progressive reduction of the sensory input.

## Overview and Take-Home Message

Cellular aging hallmarks are codependent and co-occur with the aging process. Understanding their causal network enables the conception of a framework to develop novel interventions to attenuate the aging process. As López-Otín et al. (1) referred in their review, cellular hallmarks may have beneficial or deleterious effects and may be subclassified into three main categories: primary (genomic instability, telomere attrition, epigenetic alterations, and proteostasis loss), antagonistic (deregulated nutrient sensing, mitochondrial dysfunction, and cellular senescence), and integrative (stem cell exhaustion and altered intercellular communication) hallmarks. The primary hallmarks are the initiating triggers and are always associated with deleterious effects, such as DNA damage from chromosomal aneuploidies, mtDNA mutations, telomere loss, epigenetic drift, and defective proteostasis. On the opposite, the antagonistic hallmarks have a beneficial responsive effect to attenuate damage when present in lower levels; however, when these are exacerbated or present at chronic levels, especially when promoted by the primary hallmarks, they have a progressive harmful effect, inducing cellular damage and promoting the aging process. The integrative hallmarks result from the accumulated damage from the primary and antagonistic hallmarks and directly interfere with tissue homeostasis and account for age-associated functional decline.

To face the increase in average life expectancy, many therapeutic interventions aiming at the life-span expansion have emerged (1). Nevertheless, many of these therapeutic interventions comprise expensive pharmacologic agents associated with an increased complication risk because of adverse events and polymedication. On the other hand, physical exercise is free, reduces the risk of many potentially lethal diseases, and helps strike the increasing sedentary behavior and physical-inactivity pandemic. Within this line, although exercise does not mitigate the aging process, it attenuates many of the deleterious systemic and cellular effects and improves the function of most of the mechanisms involved in aging. In this sense, further research on its most effective benefits in elderly people is warranted.

Looking at the big picture, although many paths lead to Rome, the safest and most triumphant route should extensively rely on physical exercise. This should be seen as a polypill, and the elderly community should be encouraged to engage in the continuous and regular practice of healthy physical activities. The motto is “Move for your life,” and remember, exercise is medicine.

## Author Contributions

AR-M, AL, and RA conceived the presented idea, performed the literature review and drafted the manuscript. CR, AM-P, FC, and JE-M provided advice on key aspects of the manuscript and critically revised the manuscript for important intellectual content. All the authors discussed the final manuscript and approved the final version.

## Conflict of Interest Statement

The authors declare that the research was conducted in the absence of any commercial or financial relationships that could be construed as a potential conflict of interest. The handling Editor declared a shared affiliation, though no other collaboration, with one of the authors RA.

## Abbreviations

ACSM, American College of Sports Medicine; Akt, protein kinase B; AMP, adenosine monophosphate; AMPK, 5’-adenosine monophosphate-activated protein kinase; ASC, speck-like protein caspase; ATP, adenosine triphosphate; AUF1, AU-binding factor 1; BDNF, brain-derived neurotrophic factor; CRP, C-reactive protein; DNA, deoxyribonucleic acid; FoxO3, Forkhead box O3; GH, growth hormone; GHRH, growth hormone-releasing hormone; Glut 4, glucose transporter type 4; GnRH, gonadotropin-releasing hormone; GTP, guanosine triphosphate; hnRNP D, heterogeneous nuclear ribonucleoprotein D; HSPs, heat-shock proteins; IGF-1, insulin-like growth factor 1; IL, interleukin; MEF2, myocyte enhancer factor-2; miRNA, microRNA; mRNA, messenger RNA; mtDNA, mitochondrial DNA; mTOR, mammalian target of rapamycin; Myf, myogenic factors; MyoD, myoblast determination protein; NAD, nicotinamide adenine dinucleotide; NF-kB, nuclear factor kappa B; NK, natural killer; NO, nitric oxide; PDK4, pyruvate dehydrogenase kinase isozyme; PGC-1α, peroxisome proliferator-activated receptor gamma coactivator 1-alpha; PI3K, class 3 phosphatidylinositol 3OH kinase; RNA, ribonucleic acid; ROS, reactive oxygen species; SABG, senescence-associated β-galactosidase; SASP, senescence-associated secretory phenotype; SIRT, sirtuin; sTNF-R, TNF soluble receptors; TERT, telomerase reverse transcriptase; TFAM, mitochondrial transcription factor; TNF, tumor necrosis factor; TRF-2, telomeric repeat-binding factor 2.
